# Advances in Therapeutics for Chronic Lung Diseases: From Standard Therapies to Emerging Breakthroughs

**DOI:** 10.3390/jcm14093118

**Published:** 2025-04-30

**Authors:** Kyle D. Brewer, Niki V. Santo, Ankur Samanta, Ronjon Nag, Artem A. Trotsyuk, Jayakumar Rajadas

**Affiliations:** 1 Advanced Drug Delivery and Regenerative Biomaterials Laboratory, Cardiovascular Institute, Stanford, CA 94304, USA; kdbrewer@stanford.edu; 2 Department of Medicine, Stanford University School of Medicine, Stanford, CA 94305, USA; 3 Swaza, Inc., Mountain View, CA 94043, USA

**Keywords:** COPD, asthma, idiopathic pulmonary fibrosis, long COVID, bronchodilators, inhaled corticosteroids, gene therapy, stem cell therapy, artificial intelligence, biomarkers, digital health solutions

## Abstract

**Background:** The global health burden of chronic respiratory diseases, such as chronic obstructive pulmonary disease (COPD), asthma, idiopathic pulmonary fibrosis (IPF), and acute respiratory distress syndrome (ARDS) affects billions of people and is associated with high levels of healthcare expenditure. Conventional therapies (bronchodilators and corticosteroids) provide symptomatic benefit but take no effect on disease progression, demonstrating the need to develop new therapies. Emerging therapies treat the underlying mechanisms of these chronic diseases, which provide symptomatic relief and benefit the underlying disease. **Methods:** This review assesses the evolution of therapeutic interventions for chronic lung diseases from a series of established inhaled combination therapies to biologics, gene therapy, and even AI-based stratification of therapies for patients. In addressing these issues, we review the mechanisms of action, evidence of efficacy, and clinical trial evidence, while discussing access issues affecting the implementation of these therapies and ethical issues in relation to their use. **Results:** The review highlights recent developments in treatment approaches, such as gene therapies aimed at cystic fibrosis mutations, advanced drug delivery pathways for more accurate targeting, and stem cell-based therapies designed to replace damaged lung tissue. These developments have the potential to improve outcomes for chronic lung diseases, but the challenges, including a lack of access, adequate patient selection, and long-term safety, need to be addressed. **Conclusions:** New therapies offer tremendous potential, but their transition from laboratory to clinic still face numerous barriers including access, regulation, and a need for personalized therapy approaches. The review indicates that future research should develop strategies to reduce barriers to access, improve distribution, and improve clinical guidelines to successfully implement these new therapies.

## 1. Introduction

Chronic respiratory diseases impose substantial global burdens, not only in terms of mortality and healthcare costs but also by severely diminishing quality of life. An estimated 545 million patients suffer from chronic obstructive pulmonary disease (COPD) [1] and approximately 339 million have asthma globally [2]. Idiopathic pulmonary fibrosis (IPF) is particularly devastating, with a median survival of only 2 to 5 years [3,4], while acute respiratory distress syndrome (ARDS) accounts for nearly 10% of intensive care unit (ICU) admissions worldwide [5]. In addition, patients with asthma experience a reduction in life expectancy of about 9 years [6], and in the United States, COPD-related costs exceed USD 50 billion annually, largely due to hospitalizations [7].

Beyond these economic and mortality impacts, the clinical burden of chronic lung disease is evident in the persistent symptoms that affect daily living. Patients with COPD, asthma, IPF, and ARDS commonly experience recurrent dyspnea, chronic productive cough, and reduced exercise tolerance, all of which limit daily activities and contribute to psychological distress [1,2]. In asthma, uncontrolled dyspnea and frequent exacerbations often lead to hospitalizations and reduced life expectancy [6]. Similarly, in COPD, progressive airflow limitation and recurring exacerbations result in disabling dyspnea and fatigue that reduce functional capacity [8,9]. In IPF, persistent dry cough and worsening dyspnea are closely linked to poorer clinical outcomes [3]. Collectively, these factors highlight the pressing need for innovative, patient-stratified therapies that not only modify disease progression but also alleviate symptom burden (Table 1).

**Figure 1:**
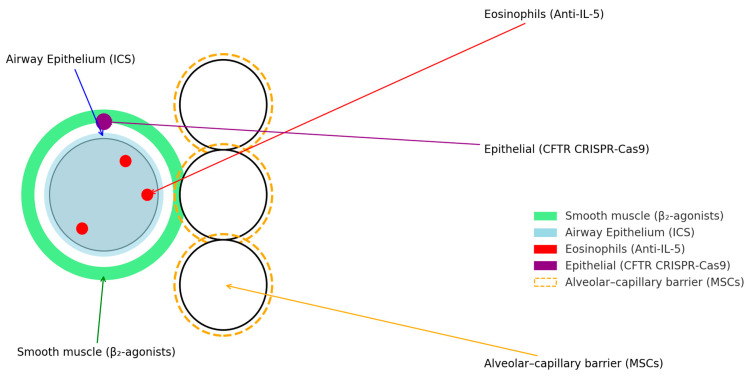
Targets of Current and Emerging Therapies in Lung Diseases.

There are three key reasons to reconsider our current treatment approaches. First, conventional pharmacologic strategies are approaching their biological limits; for instance, long-acting bronchodilators reduce COPD exacerbation frequency only by approximately 26% [8], and inhaled corticosteroids (ICS) achieve a reduction of only about 25% [9]. Second, the advent of biologics, evidenced by a roughly 50% reduction in severe asthma exacerbations [10], is now being investigated for fibrotic lung diseases such as IPF [11,12]. Third, emerging data reveal that environmental exposures, including wildfire-derived particulate matter and urban air pollution, are significant modifiers of respiratory risk, contributing to increased incidence and earlier onset of these conditions [13,14]. Recent technological advances, from repurposed small-molecule drugs to CRISPR-engineered cell therapies and artificial intelligence (AI)-assisted drug discovery platforms, are actively being explored to optimize existing pharmacologic interventions and to develop novel treatment options (Figure 1) [15,16].

Recent data highlight the necessity of considering post-COVID-19 condition (long COVID) within chronic respiratory disease epidemiology. More than one third of people with COVID-19 have persistent symptoms for greater than three months following a first infection, breathlessness a frequent symptom [17]. The pathophysiology behind such respiratory complications remains poorly explained but may be due to pulmonary fibrosis, microvascular injury, and autonomic dysfunction. With the risk of exacerbating comorbid conditions such as asthma and COPD, long COVID presents new challenges in disease management [18]. Pulmonary rehabilitation and individual breathing techniques have been helpful in alleviating dyspnea among such patients [16]. In the upcoming years, identifying biomarkers for susceptibility to long COVID and tailoring treatment to address its heterogeneous presentations will become a requirement [19,20].

## 2. Current Standard Therapies

### 2.1. Pharmacological Approaches

Bronchodilators remain the cornerstone of COPD management. Beta-agonists and anticholinergics can improve forced expiratory volume (FEV_1_) by approximately 16%, acting via the cyclic adenosine monophosphate (cAMP)-mediated smooth muscle relaxation [21,22]. Methylxanthines (e.g., theophylline) continue to be used in low-resource settings due to their low cost despite their narrow therapeutic index [23]. Inhaled corticosteroids (ICS) decrease asthma exacerbations by as much as 50–90% in mild-to-moderate disease; however, systemic absorption can lead to adverse effects such as adrenal suppression, particularly in patients with long-term use and steroid-resistant inflammation [24]. Triple therapy using long-acting β-agonists (LABA), long-acting muscarinic antagonists (LAMA), and ICS has demonstrated synergistic efficacy and may be the most effective option in moderate/severe COPD patients, with studies reported improved survival [25]. Inhaler adherence remains a challenge, as many studies show suboptimal compliance in real-world settings [26]. It is necessary to mention that bronchodilators can provide symptomatic relief but not change the underlying course of COPD over time [27]. The regular long-term use of systemic corticosteroids carries significant morbidity risks, such as osteoporosis and elevated risk of infections [28].

### 2.2. Biological Agents

Monoclonal antibody therapies have transformed the treatment of severe asthma by targeting pathways such as immunoglobulin E (IgE) and interleukin-5 (IL-5) [29]. However, their benefits in COPD remain limited, and cost considerations continue to pose challenges. In idiopathic pulmonary fibrosis (IPF), standard therapies such as pirfenidone and nintedanib remain in use, as several biologic candidates have yet not met primary endpoints in clinical trials [30]. A predominant concern in evaluating biologics is patient variability. Some patients show marked improvement, while others have little to no benefit. This variability has prompted efforts to develop predictive biomarkers to assist with the patient selection process [31].

### 2.3. Non-Pharmacological Interventions

In addition to optimized pharmacologic therapy, non-pharmacologic therapies are critical in managing chronic lung diseases, especially in patients with severe or refractory symptoms (Table 2). Pulmonary rehabilitation, oxygen therapy, and self-management education remain the mainstays of treatment, while novel device-based therapies are increasingly being explored [32,33]. Among these, bronchial thermoplasty and other electrotherapies offer promising avenues for symptom control and disease modification [34].

#### 2.3.1. Bronchial Thermoplasty

Bronchial thermoplasty is a less invasive procedure used to treat severe asthma. It employs controlled radiofrequency energy delivered via a bronchoscope to heat the airway walls, thereby reducing the mass of airway smooth muscle [34]. The reduction in the smooth muscle limits the airway’s ability to constrict during exacerbations, which in turn reduces the frequency and severity of asthma attacks. The treatment is typically performed in three sessions targeting different lung regions over a few weeks [35]. Clinical trials have established that bronchial thermoplasty can significantly improve symptom management, quality of life, and exacerbation rates in subjects with severe, refractory asthma. Its use, however, is limited by factors such as expense, the need for special equipment and expertise, and careful patient selection. Although the procedure is generally well tolerated, it is often followed by temporary respiratory worsening and post-procedure discomfort [34]. The long-term effectiveness of bronchial thermoplasty is still uncertain. Early studies showed promise with bronchial thermoplasty, but subsequent studies for long-term effectiveness lack a control group and need further study [36].

#### 2.3.2. Targeted Lung Denervation

Targeted lung denervation is a novel electrotherapy that uses radiofrequency energy to ablate pulmonary vagal nerve branches [37]. By interrupting neural circuits responsible for airway hyperresponsiveness and mucus hypersecretion, this treatment aims to reduce bronchoconstriction and inflammation, particularly in COPD patients [38]. Early clinical trials have shown promising results with improved symptom control and a reduction in the frequency of exacerbations [37]. Like bronchial thermoplasty, successful outcomes depend on appropriate patient selection and technical competence [39]. Nonetheless, there are contradictory data about the efficacy of targeted lung denervation in those with COPD. There are several studies that show significant changes in patients’ symptoms and exacerbation rates, while some other studies show little to no benefit [40]. These data are difficult to explain, but the results may be partly explained by differences in patient populations, procedural approaches, and research designs [39,40].

#### 2.3.3. Other Neuromodulatory Approaches

Other investigational electrotherapies include techniques that modulate neural activity through electrical stimulation or ablation [41,42,43,44,45,46]. These methods are designed to alter autonomic control of the airways, thereby reducing inflammation and bronchospasm. Although still in early stages, such approaches could complement existing treatments, especially in patients exhibiting marked neural dysregulation as part of their disease phenotype [47,48,49].

## 3. Emerging Therapeutic Innovations

The lung epithelium, the key protective barrier that guards against environmental insult, possesses a remarkable capacity for repair and regeneration following damage [50]. While normally quiescent, progenitor cells of the epithelium—such as airway basal stem cells and distal lung alveolar progenitors—are capable of very rapidly becoming activated following damage and re-establishing tissue integrity by lineage-restricted differentiation [50,51]. Experimental models and clinical observations have isolated certain regenerative pathways, but maladaptive repair with persistent metaplastic states and fibrotic remodeling is a frequent result of chronic injury, as in COPD, pulmonary fibrosis, and long COVID [52,53]. Stimulation of lung regeneration by stem cell-based therapy, gene editing, and selective modulation of repair signaling pathways is an exciting frontier in respiratory medicine, with possible therapeutic strategies to be employed in combination with pharmacologic and biologic therapies [54,55].

### 3.1. Small Molecules

Next-generation small molecules are being developed to target specific inflammatory and fibrotic pathways. For instance, NLRP3 (NOD-like receptor family, pyrin domain containing 3) inflammasome inhibitors are under active investigation for their potential to reduce lung inflammation [56]. Autotaxin inhibitors have shown antifibrotic promise in preclinical models [57]. In addition, inhibitors of the epithelial sodium channel (ENaC) may help restore mucociliary clearance [58]. Small molecule induction of hypoxia has also been shown to be therapeutic in some neurological conditions, such as in Leigh syndrome mouse models [59]. Despite this, advancing successful small molecule therapies for chronic lung diseases is complicated by attaining target specificity while minimizing off target effects. Many promising preclinical candidates have failed to translate their efficacy to the clinic due to these problems [60].

### 3.2. Targeted Monoclonal Antibodies

Advances in antibody engineering have led to the development of bispecific antibodies that target multiple inflammatory pathways. Anti-interleukin-5 (anti-IL-5) and anti-immunoglobulin E (anti-IgE) agents have already demonstrated significant reductions in asthma exacerbations [61], while anti-C5a antibodies are being explored in the context of sepsis-related acute respiratory distress syndrome (ARDS) and COVID-19 [62,63]. Similarly, antibodies directed against integrins have shown potential in modulating transforming growth factor-β (TGF-β) activity in idiopathic pulmonary fibrosis (IPF) [64]. One major drawback of monoclonal antibody therapies is the high cost and accessibility for many patients [65]. Additionally, the long-term safety and immunogenicity of these drugs are yet to completely elucidated [66].

### 3.3. Gene-Based and RNA Therapies

CRISPR-Cas9 technology has been successfully applied to correct cystic fibrosis transmembrane conductance regulator (CFTR) mutations [67]. Inhaled lipid nanoparticle delivery systems are emerging as a promising platform for gene therapies targeting lung diseases [68]. RNA interference (RNAi) strategies have been used to silence viral or inflammatory drivers in respiratory conditions [69]. Studies indicate that microRNA-21 (miR-21) antagomirs can mitigate fibrogenic responses in IPF [70,71]. Clinical barriers to gene and RNAi therapies include successful and safe delivery to targeted cells, off-target effects, and an immune response [72]. Long-term studies will be needed to assess the durability and safety of these therapies.

### 3.4. Other Breakthrough Technologies

Mesenchymal stem cell (MSC) therapies have shown potential for restoring the alveolar–capillary barrier in ARDS [73]. Inhalable nanogels are being investigated as vehicles for site-specific drug delivery in fibrotic lungs [74]. Also, inhalable liquid perfluorocarbon ventilation has been evaluated as an alternative oxygenation strategy in severe ARDS [75]. While these technologies have potential, they are still considered early development, and their efficacy and safety in large-scale clinical trials have yet to be determined.

## 4. Late-Stage Therapies and Clinical Trials

Recent Phase III trials have evaluated biologic agents, such as tezepelumab, that have demonstrated significant reductions in exacerbations in patients with severe asthma [76]. Although some novel agents—for example, modulators of ion channels and novel cell-based therapies—remain in early clinical development, robust trial designs that incorporate quantitative computed tomography (CT) biomarkers and patient-reported outcomes are emerging as key tools for demonstrating long-term benefits [77]. In addition, novel drug delivery platforms are addressing historical challenges related to treatment adherence. Digital health solutions, including smart inhalers that provide real-time feedback [78], are increasingly being incorporated into clinical practice to improve both adherence and overall outcomes. Advances in safety have also been reported for next-generation biologics that minimize immunogenicity through Fc receptor modifications [79], while gene therapy vectors now use tissue-specific promoters to reduce off-target effects [80]. It is important to acknowledge that although the above points are clearly established, translating positive clinical trial results into real world effectiveness remains a challenge. Adjustments for variables such as patient heterogeneity, concurrent co-morbid illness, patient behaviors, the limitations of the healthcare system, and many other phenomena all affect the treatment outcomes [81].

## 5. Advances in Diagnostics and Their Impact on Personalized Therapy

### 5.1. Biomarkers

The identification of specific biomarkers, such as blood eosinophil levels, gene mutations, and microRNAs (e.g., microRNA-21 [miR-21]), has significantly improved the ability to phenotype patients with conditions like asthma, COPD, and IPF (Table 3). For instance, an elevated eosinophil level not only determines the reaction to inhaled corticosteroids (ICS) and biologics (e.g., anti-interleukin-5 [anti-IL-5] agents) but also serves as an indicator of increased risk for exacerbations [29,61]. In addition, thiocyanate in oral fluid also shows promise as a biomarker for CFTR [82]. As these biomarkers become integrated into routine clinical assessments, therapeutic strategies can shift from a “one-size-fits-all” approach to more individualized, patient-specific regimens. However, the widespread implementation of biomarker-based personalized therapy has been hampered by the lack of standardization of biomarker assays, the expense of the tests, and the need for the education and training of clinicians [83].

### 5.2. Advanced Imaging Techniques

High-resolution computed tomography (HRCT) and quantitative imaging biomarkers have transformed our understanding of structural lung changes. These imaging modalities enable detailed visualization of emphysema, fibrosis, and airway remodeling. Quantitative assessments of lung density and structural disease patterns closely correlate with disease progression and declining lung function [77]. This detailed imaging information guides interventions—for instance, aiding in decisions regarding lung volume reduction surgery in emphysema or antifibrotic therapy in pulmonary fibrosis—by localizing the extent and distribution of lung injury. HRCT and quantitative imaging are beneficial, but they also have a high radiation dose and price compared to routine chest X-ray [84,85]. In addition, the interpretation of sophisticated imaging is complicated and not available in some clinical settings.

### 5.3. AI-Supported Interpretation

Artificial intelligence (AI) is emerging as a transformative tool in respiratory medicine. Machine learning algorithms now assist clinicians in interpreting complex diagnostic data, from HRCT scans to electronic health records, by identifying subtle patterns that may predict disease progression or response to therapy. For example, AI-supported platforms can integrate biomarker profiles with imaging data to stratify patients, forecast exacerbation risks, and recommend individualized treatment adjustments in real-time [78,79,80,81,82,83,84,85,86,87,88]. This integrated diagnostic approach not only enhances accuracy but also supports dynamic, personalized treatment algorithms that adapt as the patient’s disease evolves. AI holds great promise for enhancing respiratory care, but it also brings ethical and regulatory issues. Matters relating to data privacy, algorithmic bias, and a reliance on AI decisions might be inappropriate in a responsible implementation plan, which must be evaluated going forward [89].

## 6. Challenges and Opportunities

### 6.1. Implementation Science and Integration Within the Clinics

While significant progress has been made in developing innovative diagnostics and treatments, their translation into routine clinical practice remains challenging. Pilot studies, pragmatic clinical trials, and quality improvement initiatives are needed to identify and overcome obstacles such as clinician training, workflow integration, and resource availability. Digital health platforms and AI-enabled solutions [78,88] offer promising approaches to achieving optimal patient stratification and real-time monitoring. Obtaining real-world evidence through these programs is critical to developing effective treatment algorithms and ensuring that innovations are sustainable beyond the clinical trial environment. One of the greatest hurdles is the inconsistency of protocols used for implementing new technologies into clinical workflows, which can lead to decreased rates of adoption and variability when these technologies are implemented [90].

### 6.2. Health Disparities in Access to Novel Therapies

Despite promising advancements, significant disparities persist in access to novel therapies. Socioeconomic, geographic, and racial/ethnic disparities may limit patient access to treatments such as biologics, gene therapies, and device-based interventions. For example, patients in rural or low-resource settings often lack access to specialty centers equipped to perform procedures like bronchial thermoplasty or targeted lung denervation. These disparities are further exacerbated by variations in health insurance plans and prior authorization requirements, disproportionately affecting disadvantaged groups [87]. Addressing these gaps requires targeted policy interventions, community-based implementation strategies, and equitable reimbursement systems to ensure that all patients can benefit from the latest therapeutic developments. Targeted policy interventions, community-based implementation strategies, and fair reimbursement strategies may bridge these gaps to ensure patients are able to access new and emerging treatment options [91].

### 6.3. Cost-Effectiveness Considerations

The extremely high costs of the newer treatments—including biologics and gene therapies—present a significant challenge. Economic analyses should not only capture the short-term cost of the drug but also their indirect impact on healthcare resource utilization, long-term patient outcomes, and quality of life improvements. Although the initial costs may be high, evidence suggests that optimized therapy—when combined with digital health solutions and tailored care—can decrease exacerbation and hospitalization rates, ultimately lowering overall healthcare expenses [86]. Ensuring these new treatments have demonstrated that real-world cost effectiveness is needed to ensure long-term sustainability [92].

### 6.4. Collaborative Solutions

Multi-disciplinary collaboration among clinicians, health economists, policymakers, and technology developers are critical for bridging the gap between innovative treatments and their clinical implementation. Leveraging implementation science, evidence-based healthcare policies, and comprehensive cost-efficacy analyses will enable novel therapies for chronic lung diseases to both scientifically robust and practically feasible. A patient-centered, integrated system is vital for fully realizing the potential of these innovations and achieving improved outcomes for all patients, regardless of socioeconomic or geographic background.

## 7. Future Directions

### 7.1. Individualized Treatment and Precision Devices

Novel precision inhalers incorporating built-in sensors and AI-driven algorithms will soon enable continuous monitoring of patient lung function and real-time dosing adjustments [88]. By individualizing treatment to moment-to-moment needs, these devices can potentially optimize bronchodilation during periods of increased symptom burden, thereby reducing exacerbation frequency and improving day-to-day symptom control. Similarly, targeted biologics and gene therapies are moving toward individualized dosing schedules based on biomarker profiles [88,93]. This approach aims to enhance therapy effectiveness while minimizing side effects, ultimately leading to significant improvements in patients’ quality of life.

### 7.2. Advances in Digital Health and Remote Monitoring

The integration of AI-enabled diagnostic tools and telehealth platforms is set to revolutionize early detection and proactive exacerbation management. Predictive algorithms, which analyze data from electronic health records, wearable devices, and advanced imaging, can forecast exacerbations before they occur, thereby enabling timely intervention [78,93]. These proactive measures may not only reduce emergency room visits and hospitalization but also empower patients with greater perceived control over their condition.

### 7.3. Integration of Non-Pharmacological Interventions

New non-pharmacologic therapies, such as bronchial thermoplasty and other electrotherapies, are poised to complement new drug therapies. As these interventions target the neural and structural determinants of airway hyperresponsiveness [47,78], their incorporation into treatment algorithms is expected to sustain benefits in symptom control and overall functional status. By integrating these device-based therapies with individualized pharmacotherapy, clinicians can develop more comprehensive management strategies tailored to each patient’s specific needs.

### 7.4. Economic and Implementation Considerations

As new therapies continue to develop, real-world implementation and cost-effectiveness studies will be critical. Future research must not only assess clinical efficacy but also determine how these innovations can be feasibly integrated into everyday practice, especially in resource-limited settings. By establishing equitable reimbursement models and robust clinical guidelines, healthcare systems can ensure that the benefits—such as reduced symptom burden and improved quality of life—are accessible to all patients.

## 8. Conclusions and Practical Clinical Guidelines

In the evolving therapeutic landscape of chronic lung disease, these advances must be integrated into clinical, patient-centered treatment pathways. For instance, initial treatment for mild-to-moderate disease may rely on established therapies such as bronchodilators and inhaled corticosteroids (ICS), while patients with more severe symptoms or specific phenotypes—such as eosinophilic inflammation in asthma or significant airflow limitation in chronic obstructive pulmonary disease (COPD)—might benefit from the early addition of targeted biologics or combination therapy [8,24,29].

A step-up approach is recommended for patients with persistent symptoms despite ideal inhaler technique and adherence. In practice, healthcare professionals should:

- Evaluate Symptom Severity and History of Exacerbations: Use validated tools (e.g., the COPD Assessment Test [CAT] or Asthma Control Questionnaire) alongside spirometry to categorize patients;
- Stratify Patients by Biomarkers and Clinical Presentation: For example, patients with elevated blood eosinophils or imaging evidence of widespread structural lung changes can be prioritized for biologic medications or antifibrotic agents, respectively [29,61,77].
- Use Step-Up/Step-Down Strategies;
- Step-Up: In patients experiencing an increasing frequency of exacerbations or a decline in lung function despite monotherapy, escalate treatment to dual or triple therapy (e.g., LABA/LAMA/ICS in COPD or add anti-IL-5 biologics in severe eosinophilic asthma) [25,76];
- Step-Down: Conversely, once stable control is achieved—particularly in those initially treated with high-dose ICS—consider reducing therapy intensity to minimize long-term side effects without compromising symptom control [24];
- Leverage Digital Health Tools: Emerging AI-facilitated platforms can refine patient stratification, predict exacerbation risks, and guide timely treatment adjustments, serving as a valuable adjunct to routine clinical assessments [78,93].

This integrated, algorithm-driven approach not only optimizes therapy based on the individual patient profiles but also enables dynamic treatment adaptations in response to disease evolution and patient feedback. By combining established therapies with new technologies and personalized strategies, clinicians can bridge the gap between research advances and everyday clinical practice more effectively, ultimately improving patient outcomes and quality of life (Box 1).

Box 1 **Decision-Tree: Algorithm for Treatment Selection Based on Patient Characteristics and Biomarkers.**

1. Initial Assessment
  1.1. Evaluate Disease Severity
    ○ Spirometry: Measure FEV_1_ (% predicted) to classify severity (mild: FEV_1_ ≥ 80% predicted, moderate: 50–79% predicted, severe: <50% predicted) [8,25].
    ○ Clinical Symptoms: Assess dyspnea scales (e.g., mMRC), frequency of exacerbations, and chronic cough or sputum production [1,2].
    ○ Biomarker Profiling:
      · Blood eosinophil count:
        · Mild <150 cells/µL
        · Moderate: 150–300 cells/µL
        · Severe: >300 cells/µL (indicative of eosinophilic inflammation) [29,61]
      · Imaging: HRCT to identify emphysema or fibrotic changes [70].
      · Genetic Testing (if indicated): alpha-1 antitrypsin deficiency, CFTR mutations, etc. [68,82].
2. Determine Baseline Therapy
  2.1. Mild Disease (FEV_1_ ≥ 80%; eosinophils < 150 cells/µL)
    ○ Short-acting bronchodilators (SABA or SAMA) PRN for intermittent asthma/COPD [21].
    ○ Lifestyle Modifications: Smoking cessation, exercise, and pulmonary rehabilitation if symptomatic [32].
    ○ Monitoring: Reassess FEV_1_, symptom frequency, and eosinophil trends periodically.
  2.2. Moderate Disease (FEV_1_ 50–79%; eosinophils 150–300 cells/µL)
    ○ Asthma: Initiate low-to-moderate dose ICS ± LABA based on exacerbation risk and eosinophil levels [24].
    ○ COPD: Use LABA/LAMA dual therapy; consider ICS add-on if eosinophil count ≥ 150 cells/µL or ≥2 exacerbations/year [8,25].
    ○ ILD: Consider antifibrotic therapy (e.g., pirfenidone, nintedanib) plus supportive measures [30].
    ○ Monitoring: Evaluate symptom response and adjust therapy if persistent exacerbations occur.
  2.3. Severe Disease (FEV_1_ < 50%; eosinophils > 300 cells/µL or recurrent exacerbations)
    ○ Triple therapy (LABA + LAMA + ICS) for COPD with repeated exacerbations [25].
    ○ Targeted Biologics (e.g., anti-IL-5, anti-TSLP) in severe asthma with elevated eosinophils and frequent exacerbations [10,29].
    ○ Gene or Stem Cell Therapy in specialized ILD or genetic conditions (experimental/compassionate use) [51,67].
    ○ Non-pharmacological interventions (e.g., bronchial thermoplasty for severe asthma [47], lung volume reduction for emphysema) if appropriate.
3. Advanced/Refractory Disease
  3.1. Assess Suitability for Device-Based Therapies
    ○ Bronchial thermoplasty in severe, eosinophilic asthma refractory to maximum medical therapy [47].
    ○ Targeted lung denervation in advanced COPD with excessive vagal tone [61].
    ○ Lung volume reduction (surgical or bronchoscopic) for emphysema in upper lobes or significant hyperinflation [59].
  3.2. Evaluate for Lung Transplantation
    ○ In end-stage COPD, ILD, or cystic fibrosis with persistent hypoxemia, refer for transplant evaluation if standard therapies fail [37].
4. Step-Up/Step-Down Approach
  4.1. Step Up
    ○ Increase treatment intensity if uncontrolled symptoms, frequent exacerbations, or decline in FEV_1_, despite adherence and correct inhaler technique.
    ○ Add biologics in severe asthma if eosinophils > 300 cells/µL persistently and repeated exacerbations occur [29].
    ○ Consider advanced device therapies or combination biologics for non-responders [47,61].
  4.2. Step Down
    ○ If stable control is achieved for ≥3–6 months, consider tapering ICS dose or simplifying regimens to minimize side effects [24].
    ○ Monitor for any deterioration in symptoms or lung function upon de-escalation.
5. Ongoing Monitoring and Reassessment
  ○ Track changes in FEV_1_, eosinophil levels, and imaging findings.
  ○ Employ AI-assisted digital platforms for real-time risk prediction and therapy optimization [88,93].
  ○ Reassess comorbidities (e.g., cardiovascular disease, anxiety, obesity) and psychosocial factors that may influence adherence and outcomes [68].
6. Addressing Health Disparities and Cost
  ○ Evaluate patient access to advanced therapies (e.g., biologics, gene therapy) and mitigate barriers (insurance, geographic constraints) [68].
  ○ Incorporate cost-effectiveness data to guide resource allocation, especially in severe disease requiring expensive interventions [67].

Notes:

- Biomarker thresholds (e.g., eosinophils) complement spirometry and clinical symptoms to refine treatment decisions [29,61].
- Non-pharmacological measures (e.g., pulmonary rehabilitation, smoking cessation) remain essential at all stages [2,8].
- Advanced device-based interventions (e.g., bronchial thermoplasty, targeted lung denervation, gene therapy) are typically reserved for refractory cases but can offer substantial symptom relief [39,47].

**Table 1:** Comparison of Standard Therapies to Emerging Therapies.

Treatment Category	Drug/Intervention Name	Mechanism of Action	Clinical Efficacy	Major Side Effects	Development/ Approval Status	References
Standard Therapy	SABA (e.g., Albuterol)	β_2_-agonist that increases cAMP in airway smooth muscle, leading to rapid bronchodilation	Provides immediate symptom relief and transient improvement in FEV_1_	Tachycardia, tremor, hypokalemia	Approved; used as rescue medication	[21]
Standard Therapy	LABA (e.g., Salmeterol, Formoterol)	Prolonged β_2_ receptor stimulation for sustained bronchodilation	Reduces exacerbation frequency by ~26% in COPD	Palpitations, tremor; risk of pneumonia (especially with ICS)	Approved; standard maintenance therapy	[8]
Standard Therapy	ICS (e.g., Fluticasone)	Suppresses inflammatory cytokine release to reduce airway inflammation	Decreases exacerbations by 50–90% in mild-to-moderate asthma	Systemic absorption leading to adrenal suppression, osteoporosis	Approved; standard in asthma/COPD management	[24]
Standard Therapy	Oxygen Therapy	Provides supplemental oxygen to correct hypoxemia	Prolongs survival and improves exercise tolerance in severe COPD	Oxygen toxicity (if misused)	Approved; essential for advanced disease management	[43,44]
Standard Therapy	Pulmonary Rehabilitation	Multidisciplinary intervention (exercise, education, psychosocial support) to enhance lung efficiency	Improves exercise capacity, reduces dyspnea, and enhances quality of life	Transient muscle soreness and fatigue	Approved; integral part of chronic care	[45,46]
Emerging Therapy	Targeted Biologics (e.g., Benralizumab)	Monoclonal antibody targeting eosinophils to reduce inflammatory response	Up to 50% reduction in exacerbations in severe asthma; improved respiratory symptoms	Injection site reactions, potential immunogenicity	Approved for severe asthma; being evaluated in COPD	[10]
Emerging Therapy	Gene Therapy (CRISPR-based approaches)	Gene editing to correct disease-causing mutations or modulate inflammatory pathways	Preclinical data suggest potential improvements in mucus clearance and lung function	Off-target effects, immune responses	Early clinical trials/preclinical stage	[67]
Emerging Therapy	Stem Cell Therapy (e.g., MSCs)	Uses mesenchymal stem cells to regenerate and repair damaged lung tissue	Potential to restore alveolar–capillary barrier and improve lung function	Uncertain long-term risks; possible immunogenicity	Early clinical trials; experimental	[73]
Emerging Therapy	Advanced Drug Delivery Systems	Nanoparticle-based or smart inhalers that provide precise, controlled drug release and deposition	Improved drug deposition and adherence; potential enhanced efficacy	Device-related issues, local irritation	Under development; pilot systems in use	[74]
Emerging Therapy	Digital Health/AI-assisted Treatment	AI-driven platforms and smart inhalers for patient stratification, real-time feedback, and personalized therapy	May reduce exacerbation frequency and enable tailored therapy strategies	Data privacy concerns; potential algorithm bias	Pilot implementations; emerging field	[78,93]

**Table 2:** Clinical Trials Highlighting Emerging Lung Therapies.

Study Identifier/Name	Patient Population	Intervention	Comparator	Primary Outcome	Key Findings	Phase	References
Tezepelumab in Severe Asthma	Patients with severe asthma with frequent exacerbations	Tezepelumab (monoclonal antibody targeting TSLP)	Placebo/Standard care	Reduction in exacerbation rate and improvement in lung function	Demonstrated significant reduction in exacerbations (up to ~50% reduction)	Phase III	[76]
CT Biomarker-Guided Cell Therapy	Patients with progressive fibrotic lung disease	Novel cell-based therapy (e.g., mesenchymal stem cells)	Placebo/Standard care	Change in quantitative CT biomarkers and FVC improvement	Early data show potential to slow fibrosis progression using CT biomarkers	Phase II	[77]
Digital Smart Inhaler Pilot	Asthma/COPD patients with suboptimal inhaler adherence	Smart inhaler with real-time feedback	Conventional inhaler	Improvement in adherence and symptom control	The pilot data indicate enhanced adherence and potential reduction in exacerbations	Pilot/Early Phase	[78]
Next-Generation Biologics Safety Trial	Patients with severe asthma or COPD	Biologics engineered with modified Fc regions	Standard biologics	Safety and tolerability	Showed reduced immunogenicity and improved safety profile	Phase I/II	[79]
Gene Therapy with Tissue-Specific Promoter	Patients with genetic lung disease (e.g., CF or IPF)	Gene therapy vector incorporating a tissue-specific promoter	Conventional gene therapy vectors	Reduction in off-target effects and lung function impact	Preclinical/early-phase data indicate improved targeting and safety	Preclinical/ Phase I	[80]

**Table 3:** Biomarker Stratification and Clinical Presentation of Lung Disease Patients.

Biomarker	Associated Disease Phenotype	Clinical Presentation	Recommended Therapy/Intervention	Prognostic Impact	References
Blood eosinophil count	Severe eosinophilic asthma and COPD with frequent exacerbations	Patients often present with recurrent wheezing, episodic breathlessness, and evidence of eosinophilic inflammation (e.g., elevated sputum eosinophils, atopic history)	Use of ICS and anti-IL-5 biologics (e.g., benralizumab)	Higher counts predict better response to ICS/biologics; increased risk for exacerbations	[29,47]
FEV_1_ (% predicted)	Airflow limitation in COPD (reflecting disease severity)	Progressive dyspnea on exertion, exercise intolerance, chronic cough, and frequent exacerbations	Escalation to triple therapy (LABA + LAMA + ICS) or optimization of bronchodilator regimens	Lower FEV_1_ is associated with increased exacerbation rates and higher mortality	[8,25]
Quantitative CT biomarkers	Extent of emphysema/fibrosis in COPD or interstitial lung disease	HRCT reveals structural changes such as areas of low density (emphysema) or fibrotic bands/honeycombing; patients may have reduced exercise tolerance and gas exchange abnormalities	Consider targeted interventions (e.g., lung volume reduction procedures, antifibrotic therapies)	CT density and structural changes correlate with disease progression and functional decline	[77]
Genetic mutations	Genetic lung diseases (e.g., cystic fibrosis; predisposition in IPF)	Early-onset respiratory symptoms, family history of lung disease, and/or accelerated decline in lung function	Gene therapies (e.g., CRISPR-based correction) or mutation-specific treatments	Specific mutations can inform prognosis and predict response to targeted therapies	[64,67]
MicroRNA (e.g., miR-21)	Idiopathic pulmonary fibrosis with active fibrogenesis	Patients typically show progressive dyspnea, non-productive cough, and imaging evidence of fibrosis; elevated miR-21 is associated with active fibrotic processes	RNA interference or antagomir strategies targeting miR-21	Elevated levels are linked with faster disease progression and increased fibrogenesis	[70]
